# Calorie restriction protects neural stem cells from age-related deficits in the subventricular zone

**DOI:** 10.18632/aging.101731

**Published:** 2019-01-08

**Authors:** Deana M. Apple, Swetha Mahesula, Rene Solano Fonseca, Chang Zhu, Erzsebet Kokovay

**Affiliations:** 1 Department of Cell Systems and Anatomy, University of Texas Health Science Center at San Antonio, San Antonio, TX 78229, USA; 2 The Barshop Institute on Longevity and Aging Studies, University of Texas Health Science Center at San Antonio, San Antonio, TX 78229, USA

**Keywords:** neural stem cell, subventricular zone, calorie restriction, inflammation, neurogenesis, olfactory memory

## Abstract

The brain can generate new neurons from neural stem cells throughout life. However, the capacity for neurogenesis declines with age, reducing the potential for learning and repair. We explored the effects of calorie restriction, an established anti-aging intervention, on neural stem cells in the subventricular zone of young and aged mice. Calorie restriction transiently enhanced proliferation of neural progenitor cells in young, but not aged mice. However, calorie restriction prevented the age-related loss of neurogenesis in the aged brain. Calorie-restricted mice showed enhanced olfactory memory compared with *ad libitum-*fed controls, suggesting that calorie restriction can produce functional improvements in the aged brain. Calorie restriction also mitigated the age-related activation of microglia and subsequent increase in pro-inflammatory cytokines. Likewise, calorie restriction prevented increases in senescent cells normally observed in the subventricular zone in aged mice, further protecting this neurogenic niche from pro-inflammatory signals. Together, these data suggest that calorie restriction protects the subventricular zone microenvironment from age-related inflammation, thereby preserving neurogenesis into old age.

## Introduction

The adult brain can generate new neurons from neural stem cells. The process of neurogenesis occurs throughout life primarily in the dentate gyrus of the hippocampus and the subventricular zone (SVZ) [1,2]. In the rodent SVZ, neural stem cells give rise to immature neurons called neuroblasts. These neuroblasts then migrate through the rostral migratory stream to the olfactory bulb, where they integrate and mature into GABAergic and dopaminergic neurons. This process is highly regulated, and although the signals that control neurogenesis are not yet fully understood, it is known that neurogenesis declines with age, suggesting that the neurogenic signals are susceptible to age-related deficits observed elsewhere in the brain [3–6].

Calorie restriction is one mechanism by which age-related deficits may be reduced in aged animals. Calorie restriction can markedly increase mean and maximum lifespan and improve physiologic markers of health, including insulin sensitivity, body mass index, and plasma markers of cardiovascular disease [7–9]. Calorie restriction has beneficial effects in blood and muscle stem cell function, and can protect against neuronal damage in neurodegenerative models [10–12]. In the hippocampus, calorie restriction enhances proliferation of progenitor cells, although whether these newly born cells survive and mature into neurons is not clear [13–15].

In the SVZ, neural stem cells are highly organized into different compartments of the microenvironment. This organization is thought to be important in segregating signals that regulate neural stem cell action, such as quiescence, activation, proliferation, and migration [16–18]. Microglial cells, the innate immune cells of the brain, are a critical component of microenvironment [19]. Chronic inflammation is a known factor in aging, suggesting that inflammatory cells likely contribute to the development of deficits in the aging brain [19]. Cellular senescence is a phenomenon by which cellular division ceases in the aged organism, and is modifiable in laboratory models [20]. Inflammation is associated with senescence in *in vitro* models because senescent cells secrete pro-inflammatory cytokines.

Regulation of the SVZ neurogenic niche depends largely on signals received from the vascular plexus, which is positioned near the underlying striatum and presents circulating molecules and growth factors to the progenitor cells [16,21]. Neural stem cells proliferate in close association with the vasculature to receive these trophic and chemotactic signals that direct their lineage progression and migration [22–24]. Vasculature in the aged SVZ displays structural changes, including reduced density or rarefaction [19,25].

In this study, we show that calorie restriction is protective against age-related increases in senescence and microglia activation and pro-inflammatory cytokine expression in an animal model of aging. Further, these protective effects mitigated age-related decline in neuroblast and neuronal production, and enhanced olfactory memory performance, a behavioral index of neurogenesis in the SVZ. Our results support the concept that calorie restriction might be an effective anti-aging intervention in the context of healthy brain aging.

## RESULTS

### SVZ stem cell proliferation is transiently increased by calorie restriction

**Figure 1:**
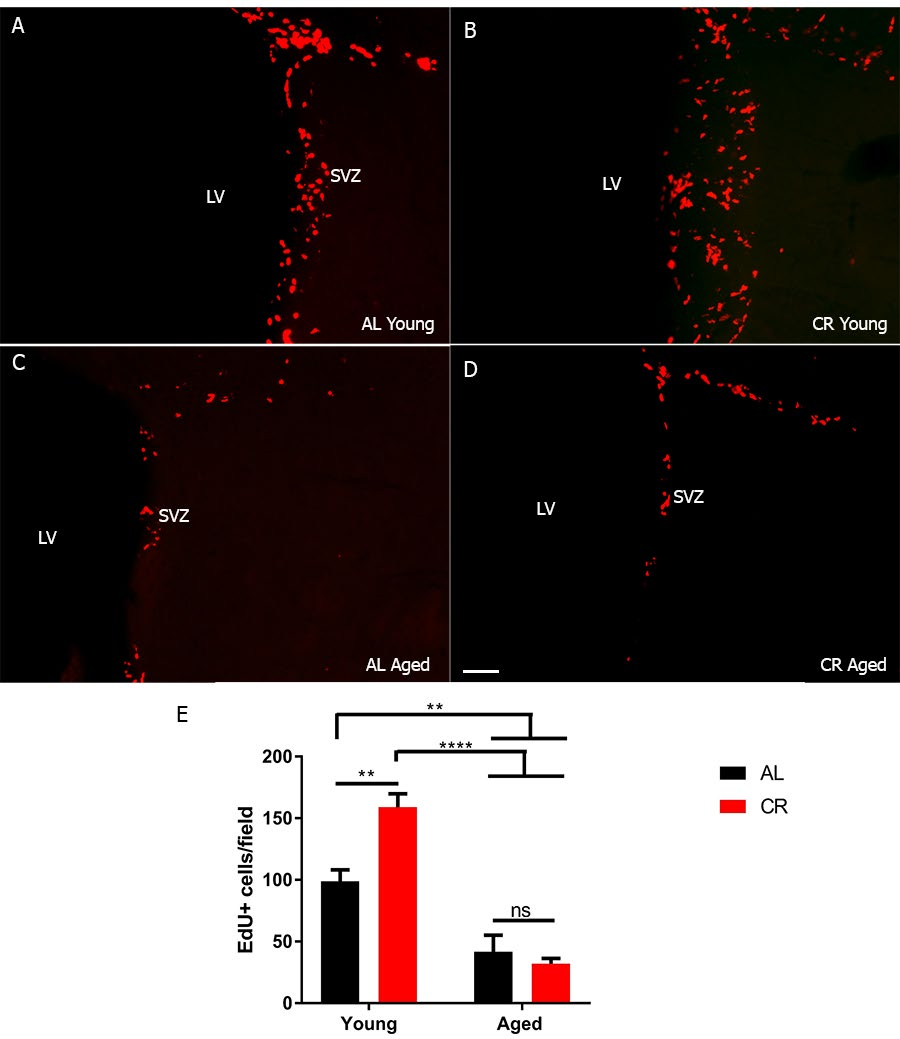
**SVZ stem cell proliferation is transiently increased by calorie restriction.** (**A-D**) Immunohistochemistry on coronal sections for EdU from young and aged brains. (**E**) Quantification of the number of proliferating cells in each group. *** = p< 0.001, ** = p<0.01, two-way ANOVA, data are presented as mean ± SEM, n=5/group. Scale bar = 50 µm.

Calorie restriction was initiated at 14 weeks of age in a stepwise fashion with an initial 10% reduction of free feeding weight followed by a 25% reduction at 15 weeks and a 40% reduction at 16 weeks. Mice were maintained at a 40% reduction until euthanized at 6 months or 12 to 18 months. Age-matched controls were fed *ad libitum*, and maintained on a free feeding diet until sacrifice. We measured numbers of proliferating cells within the SVZ of young and aged mice fed either an *ad libitum* control diet or a calorie restricted diet by injecting mice with EdU and sacrificing them 2 hours later. SVZ proliferation was transiently enhanced in young calorie-restricted (CR) mice, but declined in aged mice, regardless of their feeding paradigm (Figure 1A-D), young ad libitum (AL) diet group: 98.8 ± 9.4, young CR: 159 ± 10.9, aged AL 41.7 ± 13.5, aged CR 32 ± 4.4, p < 0.05, F (1, 12) = 11.8). We detected no differences between male and female mice. This result suggests a temporally sensitive window during which calorie restriction can impact proliferation in the SVZ of aging mice.

### Calorie restriction protects against the loss of neurogenesis in the aged SVZ

**Figure 2:**
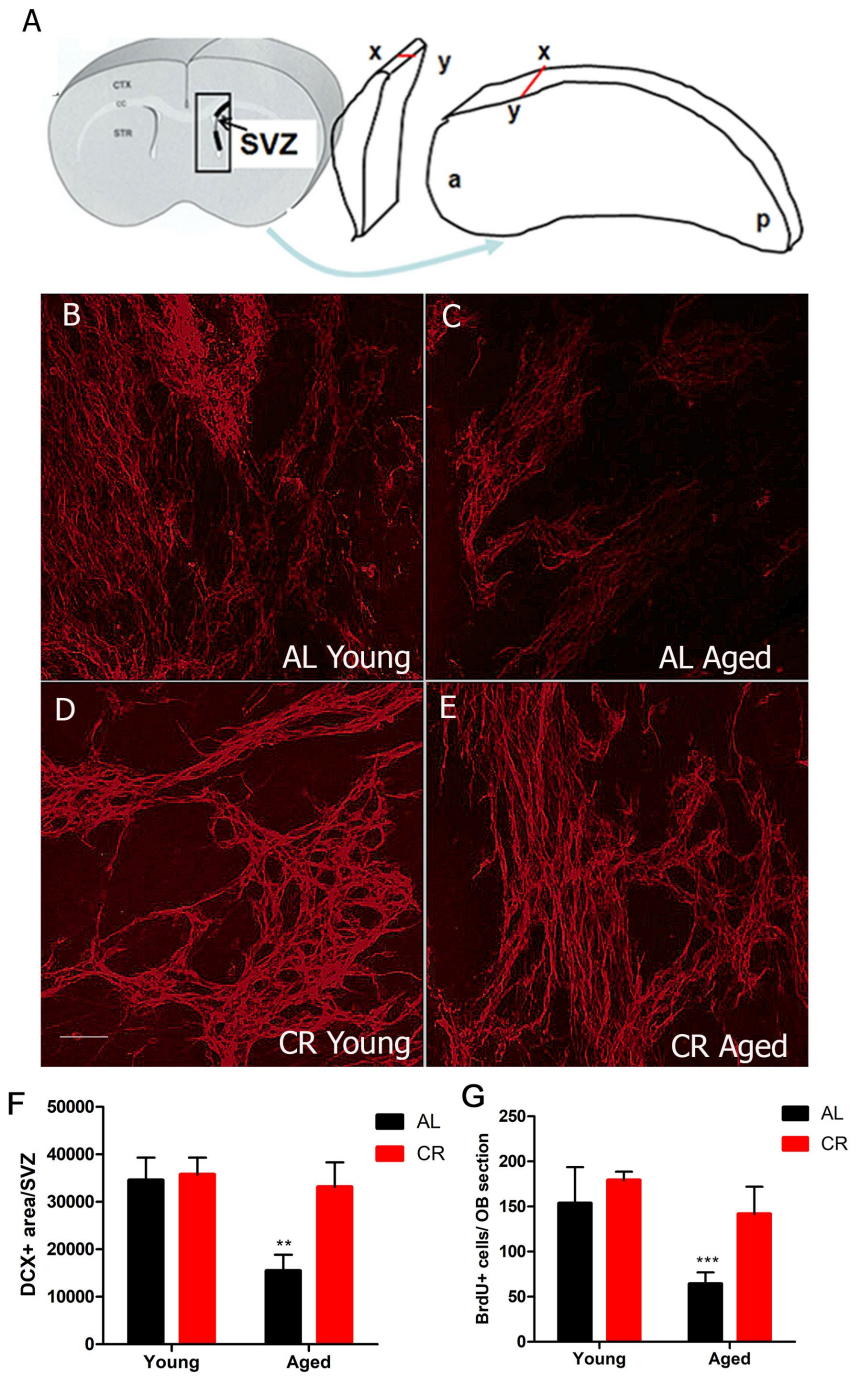
**Calorie restriction protects against the loss of neurogenesis in the aged SVZ.** (**A**) Cartoon showing the 3-dimensionally preserved SVZ wholemount. (**B-E**) Confocal projection images of immunohistochemistry for the neuroblast marker DCX performed on SVZ wholemounts in young and aged AL and CR mice. (**F**) Quantification of the amount of DCX immunostaining for the different groups. (**G**) Quantification of the number of BrdU+ cell in the olfactory bulb two weeks after BrdU injection. *** = p< 0.001, ** = p<0.01, two-way ANOVA, data are presented as mean ± SEM, n=5/group. Scale bar = 50 µm.

To begin to test whether CR preserves neuron production, we measured the number of neuroblasts (progenitor cells committed to a neuronal fate) in the SVZ. We used wholemount preparations to preserve the 3-dimensional structure of the SVZ niche for imaging studies (Figure 2). Wholemounts were immunostained for the neuroblast marker doublecortin (DCX) and imaged using confocal microscopy. As expected, we observed fewer neuroblasts in aged AL mice. However, there was no significant reduction in neuroblasts in the young and CR aged group (Figure 2A-E).

To test whether this survival of neuroblasts yielded more new neurons being born in the olfactory bulb, we measured olfactory bulb neurogenesis using the nucleotide analog BrdU, which was injected for 5 days. Mice were sacrificed two weeks later to allow BrdU-labeled cells time to reach the olfactory bulb. Based on mean numbers of BrdU-positive cells in the olfactory bulb, there were no differences in neuron production in the two groups of young mice. As expected, neurogenesis was significantly reduced in aged mice fed a normal diet. However, new neuron production was not reduced among aged mice in the CR group (Figure 2F), young AL: 153.6 ± 12.6, young CR: 179.3 ± 4.7, aged AL 64.3 ± 3.63, aged CR 141.7 ± 8.0, p < 0.05, F (1, 38) = 7.311). These data suggest that CR had no significant effect in young mice, but preserved neurogenesis in older mice.

### Olfactory memory is enhanced by calorie restriction

**Figure 3:**
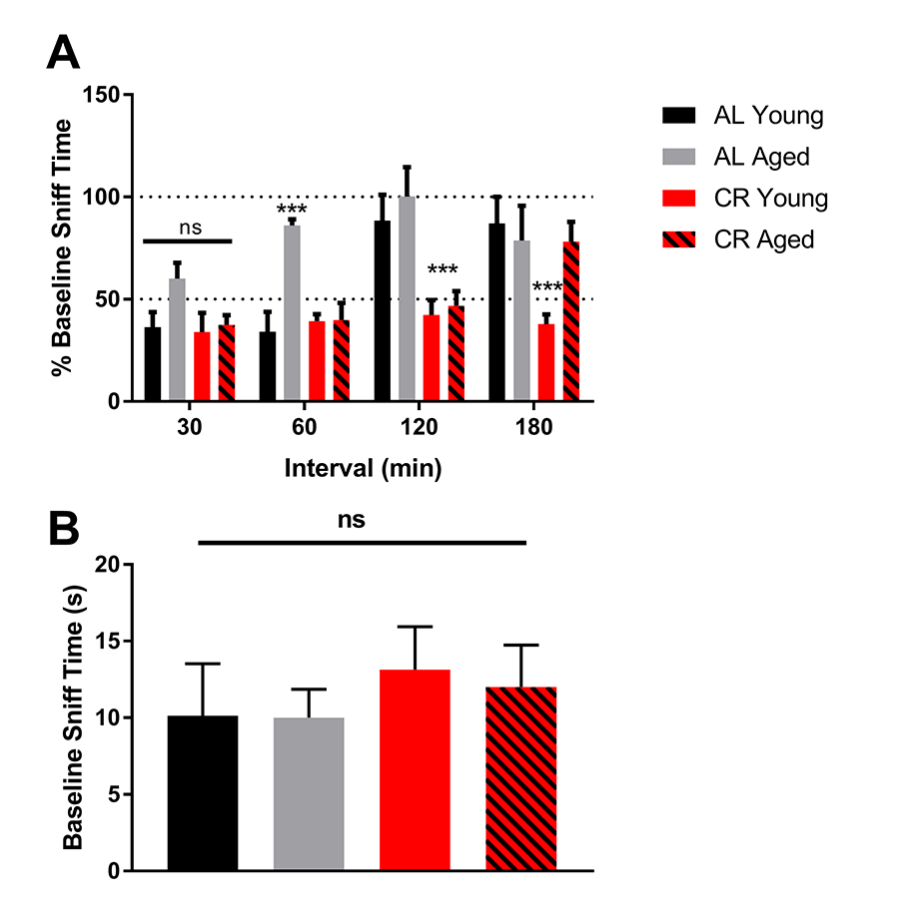
**Olfactory memory is enhanced by calorie restriction. (A)** Quantification of the percent of time mice from the initial exposure mice spent smelling the same odor at 30-minute intervals. 100% baseline sniff time was interpreted to mean that mice found the odor to be as novel as the first time they smelled it. **(B)** Quantification of the time each group spent sniffing a novel odor during the baseline trial. **** = p<0.0001, *** = p< 0.001, ** = p<0.01, * = p<0.05, two-way ANOVA, data are presented as mean ± SEM, n=10/group.

In olfactory testing, young and aged mice fed a CR diet consistently outperformed young and aged mice fed an AL diet. All groups displayed equal sniffing times during the first odor exposure across trials, and retained olfactory memory following a 30-minute interval between odor exposures. However, during a 60-minute exposure interval, aged AL mice failed to retain olfactory memory, while all other groups recalled the odor with similar accuracy. Only mice fed a CR diet recalled an odor after a 120-minute interval between odor exposures, with the aged CR group outperforming the young AL group. No mice recalled odors after a 180-minute interval (Figure 3A).

During the first 5-minute exposure, all mice, regardless of age or diet, spent the same amount of time sniffing the odor-containing probe, indicating that the baseline was similar among mice. In addition, there was no effect of novelty between trials or testing days (Figure 3B). These data indicate that olfactory memory was not only protected, it was enhanced in mice fed a CR diet.

### *SVZ* vasculature is remodeled with age despite calorie restriction

**Figure 4:**
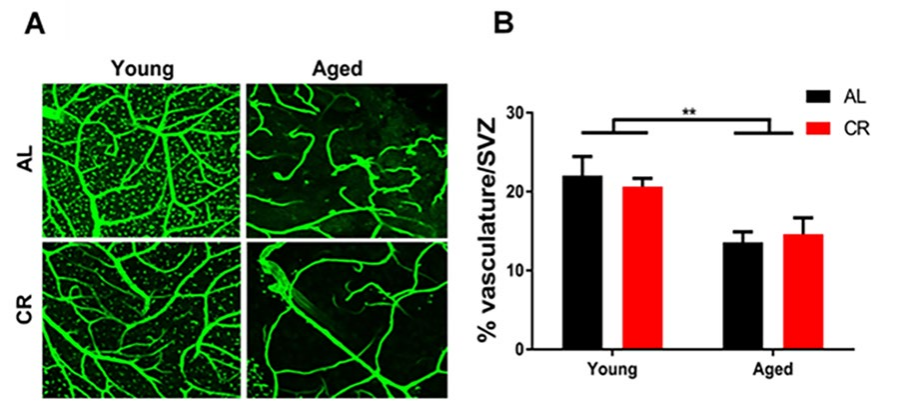
**Vascular density declines in the aged subventricular zone despite dietary intervention.** (**A**) Confocal images of the SVZ vascular plexus labeled with laminin. (**B**) Quantification of the vasculature density. ** = p< 0.01, two-way ANOVA, data are presented as mean ± SEM, n=5/group.

SVZ vascular density declined in all aged mice despite dietary intervention (Figure 4). This result suggests that the protective effects of calorie restriction are likely due to other modifiable factors in the microenvironment, such as inflammation, rather than by preserving vascular structure in the aging brain.

### Calorie restriction prevents increased inflammation and senescence within the aged SVZ

The neural stem cell microenvironment can influence stem cell activity across the lifespan. We have previously shown that the SVZ microenvironment becomes inflammatory with age, correlating with declining neural stem cell function. We also found that proliferation and differentiation potential of neural stem cells is influenced by the activation state of microglia, the immune cells of the brain [19].

**Figure 5:**
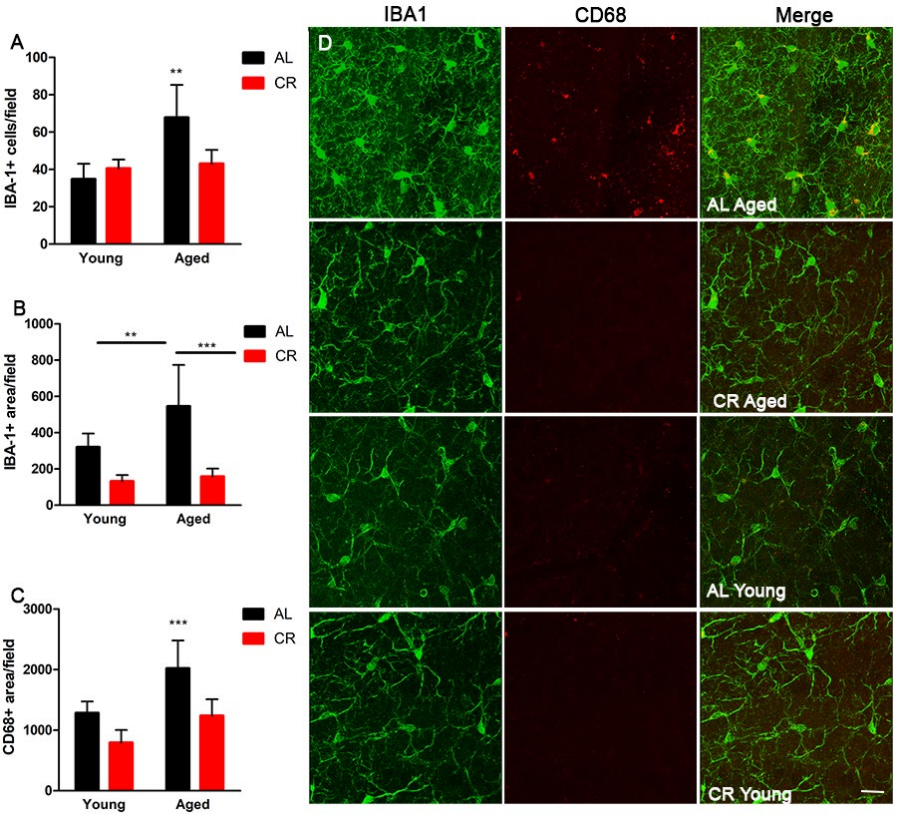
**Microglia activation is mitigated by calorie restriction.** (**A**) Quantification of the number of cells expressing the microglia marker Iba1 in the young and aged CR and AL mice. (**B**) Quantification of Iba1 reactivity in the different groups of mice. (**C**) Quantification of the activated microglia marker CD68 in the SVZ of the groups of mice. (**D**) Representative confocal images of Iba1 and CD68 microglia expression in the different groups of mice. Scale bar =25 µM. ***=p<0.001, **=p<0.01, two-way ANOVA, data are presented as mean ± SEM, n=5/group.

**Figure 6:**
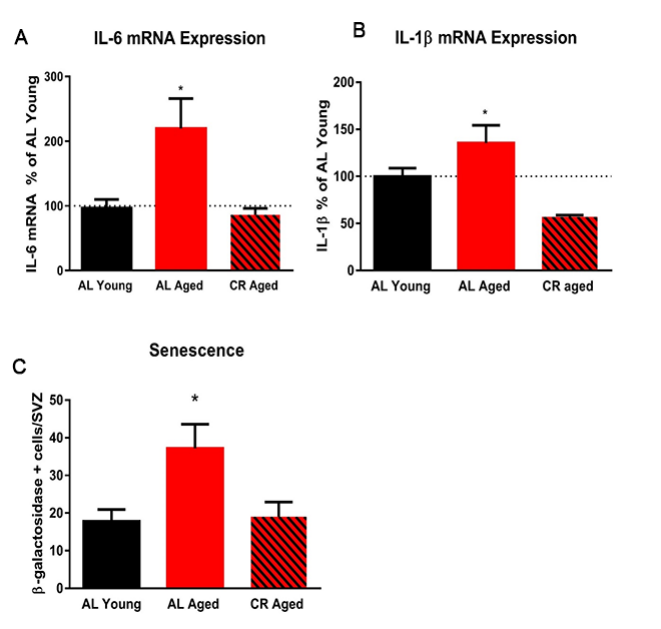
**Calorie restriction abrogates markers of inflammation in the subventricular zone.** (**A**) Quantification of the expression levels of the pro-inflammatory cytokine IL-6 in the SVZ using qRT-PCR. (**B**) Quantification of IL-1β in the SVZ. (**C**) Quantification of the number of senescent cells in the SVZ. * = p< 0.05, one-way ANOVA, data are presented as mean ± SEM, n=5/group.

To begin to investigate inflammatory markers in young and aged mice fed either AL or CR diets, wholemount SVZ preparations were immunostained for ionized calcium binding adaptor molecule 1 (IBA-1), a marker of microglia, and cluster of differentiation 68 (Cd68), an inflammation-associated glycoprotein expressed on microglia. Wholemounts were imaged using confocal microscopy and the total number of IBA-1+ microglia were counted. IBA-1+ microglia were significantly more numerous in aged AL mice relative to young AL and CR mice; however, this was not the case in aged mice on a CR diet compared to young mice (Figure 5A). IBA-1+ microglia were also larger in aged AL mice relative to young AL, young CR, and aged CR, indicating an increased inflammatory phenotype with age that was not observed in aged mice fed a CR diet (Figure 5B). Similarly, microglia expression of the activation marker CD68 was also lower in the CR mice compared to those fed an AL diet (Figure 5C). Further, levels of the inflammation-associated cytokines interleukin-6 (*IL*-6) and interleukin-1-beta (*IL*-1β) were increased in aged AL SVZ, but not in aged CR mice compared to young control mice (Figure 6A). Together, these data suggest that microglia are directly impacted by calorie restriction, and this intervention decreases the inflammatory profile in the SVZ of aged mice.

We examined the effect of calorie restriction on senescence in the SVZ of young and aged mice by measuring β-galactosidase activity. Aged mice fed *ad libitum* displayed two-fold more senescent cells in the SVZ compared with young AD controls. Calorie restriction prevented this increase in senescent cells in the aged SVZ (Figure 6C). This protective effect of calorie restriction on neural stem cells is likely due in part to reduced inflammation and senescence in the SVZ niche.

## DISCUSSION

Neurogenesis continues throughout life in the mammalian brain, but begins to decline at mid-age, compromising cognition and the brain’s ability to respond to age-related damage. Calorie restriction has been extensively studied as an anti-aging intervention, but rarely as a way to examine changes to aging neural stem cells. Park and coworkers showed that calorie restriction can increase proliferation of neural stem cells in the hippocampus, but does not result in an increase in DCX^+^ neuroblasts, suggesting that calorie restriction does not increase neuronal production by neural stem cells [15]. In contrast, others have shown no effect of calorie restriction on hippocampal proliferation, but calorie restriction does promote survival of new granular cell layer neurons [14]. Thus, the overall influence of calorie restriction on hippocampal stem cells remains unclear.

Neural stem cells from the SVZ give rise to a different population of neurons than hippocampal stem cells, are developmentally derived from a different source, and have a unique niche and regulatory signals [26]. Here we show that calorie restriction also has a differential effect on proliferation and neuron production of neural stem cells in the SVZ. Calorie restriction initiated in early adulthood prevented the age-related decline in neurogenesis, with more DCX^+^ neuroblasts in the SVZ and increased numbers of BrdU^+^ cells in the olfactory bulb of aged mice. In contrast, only young mice fed a calorie-restricted diet experienced increased proliferation of stem and progenitor cells in the SVZ, while aged mice showed a decline in this proliferation regardless of diet.

These results may seem counterintuitive because proliferation is not increased in the aged SVZ of calorie-restricted mice, despite having neurogenesis equivalent to that in young mice. The mechanism for this increased production of neurons without increased proliferation remains to be elucidated. Perhaps calorie restriction increases the survival of newborn neurons. Another possibility is that as neural stem cells age, they exhibit a switch in cell fate to astrocytes, which has been observed in the hippocampus [27]; long-term calorie restriction may promote continued production of neurons. The total number of proliferating cells drops by at least 75% in neurogenic regions of the aging brain, while a larger proportion of the remaining neural stem cells continue to proliferate [4,6,28].

Together, our data show that calorie restriction preserves the ability of neural stem cells in aged mice to differentiate into neurons *in vivo* and survive after integration into the olfactory tissue. Thus, calorie restriction appears to preserve neural stem cell function in the aged brain, possibly though mitigation of progressive inflammation typically observed in the SVZ in aging mice. It will be important for future studies to investigate if this mitigation translates to better brain repair in the aged mice following traumatic brain injury and ischemia.

The presence of neural stem cells in calorie-restricted aged mice does not necessarily equate to functional neurons that can integrate into existing brain tissue. The functional effects of hippocampal neurogenesis are quantifiable using learning and memory tests [29,30]. However, the functional readout for SVZ neurogenesis is primarily observed through measures of olfactory function [30]. Olfactory memory testing revealed that mice fed a calorie-restricted diet performed better than *ad libitum*-fed mice, regardless of age. Aged calorie-restricted mice could recall odors after longer intervals between exposures compared with aged *ad libitum*-fed mice. Interestingly, young calorie-restricted mice outperformed young *ad libitum*-fed mice in the olfactory memory test, indicating that calorie restriction not only protects against the loss of olfactory memory, but enhances olfactory function above that in young control mice. This occurs despite an apparent ceiling effect of calorie restriction to increase the number of newborn neurons in the olfactory bulb of young mice, suggesting that the neurons may be surviving and integrating more effectively under calorie restriction compared with *ad libitum*-fed mice

We have previously established that a pro-inflammatory environment prevails in the SVZ niche in aged animals, and correlates with fewer neural stem cells and reduced function of those cells [19]. Short-term inflammation induced by radiation exposure can decrease hippocampal neurogenesis in young rats [31]. Furthermore, diminished hippocampal neurogenesis in brains of young rodents following transient inflammation can be reversed by anti-inflammatory treatment [32]. However, while anti-inflammatory drugs can decrease levels of inflammatory markers in the hippocampus of aged rodents, they do not fully restore neurogenesis, suggesting that chronic inflammation causes a detrimental and permanent alteration to the neurogenic microenvironment [32].

Calorie restriction exerts potent anti-inflammatory effects in peripheral tissues and can reduce levels of circulating inflammatory factors [33]. It also decreases inflammatory responses to lipopolysaccharide exposure [34] and lowers cytokine and C-reactive protein levels in the blood of rodents, non-human primates, and humans [35,36]. In particular, IL-6, a cytokine associated with senescence, is markedly reduced in aged calorie-restricted animals compared with aged *ad libitum*-fed animals [35,37,38]. In the present study, we have observed a neurogenic microenvironment in the SVZ with far fewer activated microglia, lower cytokine expression, and decreased senescence in aged calorie-restricted mice compared with *ad libitum*-fed counterparts. Our data showing decreased inflammatory and senescent markers add to the body of literature describing the anti-inflammatory effects of calorie restriction, and extend our current knowledge to include the protective effect of calorie restriction against inflammation in the SVZ neurogenic niche.

## MATERIALS AND METHODS

### Animals

Male and female C57Bl/6 mice (aged 6-18 months) were obtained from the National Institute of Aging calorie restricted colony. Mice were divided into young (6 to 7 months old) and old (12-18 months old) groups. Calorie restricted mice received less chow in a stepwise fashion to 40% of free feeding weight by 16 weeks (10% restriction at 14 weeks, 25% restriction at 15 weeks, and 40% restriction at 16 weeks) of age. Age-matched controls were fed *ad libitum*, and maintained on this diet until sacrifice. All animals were provided continuous free access to water. Animal handling and sacrifice were performed in compliance with IACUC guidelines.

Data from male and female mice were combined for this report, since we observed no differences in key measurements, including microglia activation and neurogenesis. This strategy also allowed us to use fewer mice, since this colony of calorie-restricted mice has been discontinued and mice were in limited availability.

### SVZ wholemount dissection

SVZ tissue was dissected from the striatal wall as described previously [22]. Wholemounts were processed with immunohistochemistry as described below and positioned onto slides for further confocal analysis.

### EdU and BrdU labeling and detection

Mice were injected with EdU (10 ng/ml solution) intraperitoneally (50 mg/kg) 2 hours before they were sacrificed and perfused to assess cell proliferation. For identification of newborn cells in the olfactory bulb, mice were injected daily for 4 days with BrdU. Two weeks after the last injection, mice were perfused with cold 1x PBS followed by 4% paraformaldehyde. After cryosectioning, brain sections were permeabilized with 0.3% PBST for 45 minutes at room temperature. The sections were then incubated in 2N HCl at 37° C for one hour followed by two 5-minute washes in borate buffer. Primary and secondary incubation proceeded according to immunohistochemistry protocol. The total number of EdU or BrdU positive cells were counted on all sections containing the SVZ and olfactory bulbs and averaged across sections.

### Immunohistochemistry and measurement of senescent cells

Brain sections were fixed in 4% paraformaldehyde for 1 hour at room temperature and permeabilized/washed in 0.3% PBST. SVZ whole mounts were fixed in 4% paraformaldehyde overnight at 4°C and permeabilized/washed with 2% PBST. Cell cultures were fixed in 4% PFA for 15 minutes at room temperature and permeabilized/washed with 0.1% PBST. Prior to primary antibody incubation, tissues were incubated in blocking solution containing 10% normal donkey serum in the appropriate PBST concentration overnight at 4° C. Incubation in primary antibodies (anti-BrdU, 1:250, Novus Biologicals, #NB-500-169, Littleton, Colorado; anti-IBA1, 1:200, Wako, #019-19187, Richmond, Virginia; anti-Cd68, 1:250, ABD Serotech, #MCA1957, Oxford, UK; anti-GFAP, 1:500, Dako/Agilent, #Z0334, Santa Clara, California; anti-laminin, 1:250, Sigma Aldrich, #L9393, St. Louis, Missouri; anti-DCX, 1:200, Santa Cruz, #SC8066 Dallas, Texas; proceeded under blocking solution overnight at 4° C. The tissue was washed 3 times in PBST solution after incubation in primary antibodies. Under the same blocking solution, secondary antibodies were added and incubation proceeded overnight at 4° C. The tissue was washed 3 times in PBST solution after incubation in secondary antibodies. Nuclear staining consisted of 10 minutes of incubation in 1:1000 DAPI in PBS. Species-appropriate secondary antibodies were purchased from Jackson ImmunoResearch and used at a concentration of 1:250. The number of positive cells was measured in 10 confocal z-stacks for each mouse. Senescent cells were detected using The Calbiochem Senescence Detection Kit (EMDMillipore # QIA117) using the protocol in the kit. Beta-galactosidase positive cells were counted in the entire SVZ.

### Preparation of mRNA and real-time PCR

Subventricular zone tissues were microdissected and processed using the RNeasy mini kit for RNA purification (Qiagen, Hilden, Germany, cat. # 74104). RNA amplification was performed using a 7500 Real-Time PCR System Thermal Cycler (Applied Biosystems, Foster City, CA) with Sybr Green PCR master mix (Applied Biosystems, cat. # 4309155) for amplification detection. Primer sequences were as follows: IL-6 Fwd: CAACGATGATGCACTTGCAGA Rev:GGTACTCCAGAAGACCAGAGG, IL-1β Fwd: GAGAACCAAGCAACGACAAAATA Rev: TGGGGAACTCTGCAGACTCAAAC. The ΔΔ Ct protocol was followed for quantification as previously described [39].

### Olfactory figure

Olfactory memory is a neurogenesis-dependent process distinctly different from hippocampal-dependent memory [30]. In tests of olfactory memory, a novel, non-food odor is presented to a mouse, and sniff time is recorded across a 5-minute exposure period. The odor is removed for a time interval (30, 60, 120, or 180 minutes), and then presented to the mouse again for a second 5-minute exposure period. A relative decrease in sniff time during the second exposure indicates an olfactory memory of that odor. We assessed olfactory memory as previously described [30]. Briefly, mice were exposed to a 0.5 μl sample of a novel odor (Aura Cacia essential oils: cinnamon, clove, rosewood, lemon, thyme, or citronella, www.auracacia.com) blotted onto a 10 mm x 10 mm piece of Whatman filter paper. Each mouse was exposed to a control probe with 0.5 μl distilled water blotted onto the filter paper to familiarize the mouse with the procedure prior to the first testing session. The paper was secured in a disposable pipette and suspended in the home cage for 5 minutes, and time spent sniffing and rearing near the scented probe was recorded. Mice were exposed to the same scented probe following a randomly selected time interval (30, 60, 120, 180, or 240 minutes); sniffing and rearing times were again measured. Each mouse was tested in each time interval, with at least 24 hours between testing sessions. Different odors were used on each test interval so that no mouse was exposed to a single scent outside of the two testing sessions. Mice that did not move during the testing session were not included in the analysis.

### Statistical analysis

Differences among experimental groups were analyzed using a two-way ANOVA with a Newman-Keuls correction for multiple comparisons. Between-group analyses were performed using a one-way ANOVA with a Bonferroni correction for multiple comparisons. All comparisons utilized an alpha value of 0.05.
